# GSK-3β at the Crossroads in Regulating Protein Synthesis and Lipid Deposition in Zebrafish

**DOI:** 10.3390/cells8030205

**Published:** 2019-02-28

**Authors:** Yaqi Gu, Lili Gao, Qiang Han, Ao Li, Hairui Yu, Dongwu Liu, Qiuxiang Pang

**Affiliations:** 1 Anti-aging & Regenerative Medicine Research Institution, School of Life Sciences, Shandong University of Technology, Zibo 255000, China; 13110701052@stumail.sdut.edu.cn (Y.G.); gaoliazdy11@163.com (L.G.); acqx@163.com (A.L.); 2 Sunwei Biotech Shandong Co., Ltd., Weifang 261205, China; hq0536@dingtalk.com; 3 College of Biological and Agricultural Engineering, Weifang Bioengineering Technology Research Center, Weifang University, Weifang 261061, China; yuhr@wfu.edu.cn; 4 School of Agricultural Engineering and Food Science, Shandong University of Technology, Zibo 255000, China

**Keywords:** GSK-3β, protein synthesis, lipid deposition, muscle, zebrafish

## Abstract

In this study, the mechanism by which GSK-3β regulates protein synthesis and lipid deposition was investigated in zebrafish (*Danio rerio*). The vector of pEGFP-N1-GSK-3β was constructed and injected into the muscle of zebrafish. It was found that the mRNA and protein expression of tuberous sclerosis complex 2 (TSC2) was significantly increased. However, the mRNA and protein expression of mammalian target of rapamycin (mTOR), p70 ribosomal S6 kinase 1 (S6K1), and 4E-binding protein 1 (4EBP1) was significantly decreased by the pEGFP-N1-GSK-3β vector in the muscle of zebrafish. In addition, the mRNA and protein expression of β-catenin, CCAAT/enhancer binding protein α (C/EBPα), and peroxisome proliferators-activated receptor γ (PPARγ) was significantly decreased, but the mRNA expression of fatty acid synthase (FAS), acetyl-CoA carboxylase (ACC), ATP-citrate lyase (ACL), and HMG-CoA reductase (HMGCR) was significantly increased by the pEGFP-N1-GSK-3β vector. The activity of FAS, ACC, ACL, and HMGCR as well as the content of triglyceride (TG), total cholesterol (TC), and nonesterified fatty acids (NEFA) were significantly increased by the pEGFP-N1-GSK-3β vector in the muscle of zebrafish. The content of free amino acids Arg, Lys, His, Phe, Leu, Ile, Val, and Thr was significantly decreased by the pEGFP-N1-GSK-3β vector. The results indicate that GSK-3β may participate in regulating protein synthesis via TSC2/mTOR signaling and regulating lipid deposition via β-catenin in the muscle of zebrafish (*Danio rerio*).

## 1. Introduction

As a highly conserved serine/threonine protein kinase, glycogen synthase kinase-3 (GSK-3) is encoded by two genes, which generate two protein homologs GSK-3α and GSK-3β [1,2]. It is known that GSK-3β plays a significant role in Wnt signaling pathway [1]. In the resting cells, GSK-3β binds with adenomatous polyposis coli (APC), Axin-scaffolding protein, and β-catenin [3], which lead to β-catenin phosphorylation and subsequent proteosome-mediated degradation [4,5]. However, following Wnt ligands’ binding with the LRP5/6 and Frizzled co-receptors, GSK-3β phosphorylates LRP5/6 and Axin complex is depolymerized. Thus, GSK-3β could not phosphorylate β-catenin, which resulted in the accumulation of β-catenin [6]. Then β-catenin translocates to the nucleus and regulates the transcription of a series of target genes by binding to the family of LEF/TCF [7]. 

The mammalian target of rapamycin (mTOR), a conserved protein from yeast to humans, regulates metabolism and cell growth through mTOR complex 1 (mTORC1) and mTOR complex 2 (mTORC2) [8,9]. In mammals, mTORC1 regulates protein synthesis via the target of rapamycin (mTOR), Deptor and PRAS-40, and raptor (regulatory associated protein of mTOR) [10]. The eukaryotic initiation factor 4E binding protein 1 (4EBP1) can be phosphorylated by mTOR, which results in the release of eukaryotic initiation factor 4E (eIF-4E). Moreover, the ribosomal protein S6 kinase 1 (S6K1) could be phosphorylated by mTOR and results in the phosphorylation of ribosomal protein S6 (rpS6) [11]. S6K1 and eIF-4E are closely related to mRNA translation and protein synthesis [11,12]. In addition, mTORC2 mainly consists of mTOR, mSin1, Rictor, Deptor, and Protor-1, which elicit distinct biological functions from mTORC1 [13,14]. mTORC2 is largely insensitive to rapamycin and directly phosphorylates S6K1 [15].

GSK-3β participates in numerous biological processes, including cell differentiation, cell cycle, and apoptosis [1,16]. The deregulation of GSK-3β is related to the development of diabetes, cancer, neurodegenerative disease as well as bipolar disorder [17]. In addition, GSK-3β plays a significant role in regulating TSC2/mTOR signaling, which is involved in regulating cell growth [18,19]. Until recently, the function of GSK-3β on regulating protein synthesis and lipid deposition was unknown in fish species. It is interesting to investigate whether GSK-3β participates in regulating protein synthesis and lipid deposition in zebrafish. In this study, the GSK-3β gene was overexpressed to investigate the mechanism that GSK-3β participates in regulating protein synthesis and lipid deposition in zebrafish (*Danio rerio*). 

## 2. Materials and Methods

### 2.1. Animals and Experimental Conditions

Zebrafish (AB strain), 6 months of age (~3.4 cm), were cultured in dechlorinated water (28 °C) at a light:dark photoperiod (12 h:12 h) in flow-through tanks. Zebrafish were acclimated for 15 days and fed twice daily with a commercial diet (Sanyou Beautification Feed Tech Co., Ltd.,Beijing, China). Animal experiments were approved by the Animal Ethics Committee of Shandong University of Technology in accordance with the Guidelines for Proper Conduct of Animal Experiments (Science Council of China).

### 2.2. Construction of the pEGFP-N1-GSK-3β Vector

**Figure 1:**
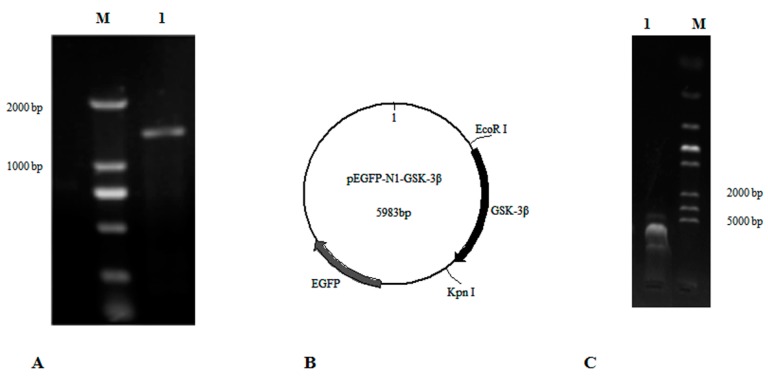
The construction of pEGFP-N1-GSK-3β. (**A**) The ORF of GSK-3β was cloned according to primers GSK-3β-F1 and GSK-3β-R1. Lane 1, DNA marker; Lane 2, GSK-3β. (**B**) The map of pEGFP-N1-GSK-3β. (**C**) The connected fragment of GSK-3β and pEGFP-N1 was amplified using PCR. Lane 1, the connected fragment of GSK-3β and pEGFP-N1; Lane 2, DNA marker.

GSK-3β belongs to the PKc-like super family, and does not have transmembrane regions, based on an analysis with TRMHMM server v2.0. The open reading frame (ORF) of GSK-3β (GenBank: NM_131381.1) was analyzed and cloned with the primers GSK-3β-F1 and GSK-3β-R1 (Table 1, Figure 1A). Then the ORF of GSK-3β with the restriction enzyme sites (*EcoR* I and *Kpn* I) was cloned with the primers GSK-3β-F2 and GSK-3β-R2 (Table 1). Finally, a fragment of GSK-3β was inserted into the plasmid pEGFP-N1 by using the double-enzyme cleavage method (Beijing TransGen Biotech Co. Ltd, Beijing, China) (Figure 1B,C). This new vector was designated as pEGFP-N1-GSK-3β and transformed into *E. coli* DH5a for plasmid amplification.

### 2.3. Injection of the pEGFP-N1-GSK-3β Vector

Seventy-two zebrafish were randomly distributed into twelve glass tanks. The vector of pEGFP-N1-GSK-3β (500 ng dissolved in 10 μL PBS) was injected into the muscle of each fish in the experimental group (6 tanks) according to a method by Hansen et al. [20]. In addition, each fish in the control group (6 tanks) received 10 μL PBS. Six days later, after the fish were anesthetized with 0.1 g/L MS222, 6 independent muscle samples were collected from the control and experimental groups, respectively. Each sample consists of muscle from three fish. The samples were frozen in liquid nitrogen and stored at −80 °C for molecular biology analysis. Moreover, the other 6 muscle samples were collected and homogenized in cold saline for biochemical analysis. For the control group, the fish were sampled as the experimental group.

### 2.4. RNA Extraction and Real-Time Quantitative Polymerase Chain Reaction

Total RNA was extracted from muscle samples using Trizol reagent (Invitrogen, Carlsbad, CA, USA). Then 1.0 μg total RNA was subjected to reverse transcription with reverse transcribed to cDNA by PrimeScript™ RT Reagent Kit (Takara Bio., Inc., Otsu, Japan), and SYBR^®^ Premix Ex Taq™ II was used to quantify the expression level of genes (Takara, Japan). The primer sequences for GSK-3β, TSC2, mTOR, S6K1, 4EBP1, β-catenin, C/EBPα, PPARγ, fatty acid synthase (FAS), acetyl-CoA carboxylase (ACC), ATP-citrate lyase (ACL), HMG-CoA reductase (HMGCR), and reference gene (β-actin) were designed and listed in Table 2. Real-time PCR was carried out using Roche Lightcycler480 (Roche Instrumnet Center AG, Rotkreuz, Switzerland). The 2^−ΔΔCT^ method was employed to analyze the differences of relative gene expression in each sample using β-actin as the internal reference gene [21]. 

### 2.5. Western Blot Analysis

Protein was extracted from muscle samples with Protein Extraction Kit (Beyotime Biotechnology, Wuhan, China), and protein concentration was measured using BCA Protein Assay Kit (Beyotime Biotechnology, Wuhan, China) [22]. After gel electrophoresis and transfer to polyvinylidene difluoride membranes, the membranes were incubated with 5% skimmed milk for 1 h at room temperature. Then membranes were probed with the primary antibody and the appropriate HRP-conjugated secondary antibody. Antibodies directed against β-catenin (Cat. No. 8480, 1:1000), GSK-3β (Cat. No. 12456, 1:1000), PPARγ (Cat. No. 2435, 1:1000), C/EBPα (Cat. No. 8178, 1:1000), TSC2 (Cat. No. 4308, 1:1000), S6K1 (Cat. No. 2708, 1:1000), 4EBP1 (Cat. No. 4923, 1:1000), and FAS (Cat. No. 3180, 1:1000) were purchased from Cell Signaling Technology Inc. Antibody against mTOR (Cat. No. GTX124771, 1:1000) was purchased from GeneTex, Inc. β-actin (sc-1615, 1:10,000) was purchased from Santa-Cruz Inc. Densitometry analyses were performed with the Image J software (National Institutes of Health, Bethesda, MD, USA).

### 2.6. Assay the Content of Biochemical Index Related to Lipid Deposition in the Muscle

The samples of muscle were homogenized and the supernatants were collected for biochemical analysis after centrifugation at 4000× *g* for 10 min, respectively. The content of triglyceride (TG) and total cholesterol (TC) was assayed by enzymatic colorimetric methods (GPO-PAP for triglycerides and CHOD-PAP for cholesterol) according to instructions provided with the TG and TC kits. As copper ion could bind to nonesterified fatty acids (NEFA), NEFA content was analyzed by detecting the content of copper ion according to procedures described in the NEFA assay kit. FAS activity was assayed following decrease in absorbance at 340 nm resulting from the oxidation of NADPH dependent on malonyl-CoA, according to procedures mentioned in the FAS kit. The activity of GSK-3β was measured using the GSK-3β Activity Assay Kit (Sigma/Aldrich, St. Louis, MO, USA) following product instructions. The assay was based on immunoprecipitation of GSK-3β using a specific anti-GSK-3β antibody. The immunoprecipitated kinase was incubated with γ-^32^P-ATP and incorporation of ^32^P into the substrate was measured. ACC was able to catalyze acetyl coenzyme A, NaHCO_3_ and ATP into malonyl coenzyme A, ATP, and inorganic phosphorus. The activity of ACC was determined based on the increasing amount of inorganic phosphorus, which was detected at 660 nm by colorimetric measurement of phosphorus as molybedenum blue. One U means that 1 mg protein produces 1 μmol inorganic phosphorus per hour. In the presence of ATP and coenzyme A, ACL catalyzes citric acid into acetyl coenzyme A, oxaloacetic acid, and adenosine diphosphate. Malic dehydrogenase further catalyzes oxaloacetic acid and NADH to produce malic acid and NAD^+^. The activity of ACL was assayed at 340 nm based on its ability to utilize NADH, and one U means that 1 mg protein consumes 1 nmol/L of NADH per min. The activity of HMG-CoA reductase (U/mg protein) was assayed at 340 nm based on its ability to utilize NADPH using HMG-CoA as substrate, and one U means that the enzyme utilizes 1 nmol/L of NADPH per min. The kits of TG, TC, NEFA, and ACC were purchased from Nanjing Jiancheng Bioengineering Institute (Nanjing, China), and the kit for FAS activity was purchased from Beijing Solarbio Science & Technology Co., Ltd. (Beijing, China). In addition, the ACL and HMGCR kits were purchased from Shanghai Yaji Biological Co., Ltd. (Shanghai, China). Finally, the coomassie brilliant blue G250 staining method was used to determine protein concentration of the supernatants.

### 2.7. Detection the Content of Free Amino Acid in the Muscle of Zebrafish

According to a method by Dambergs et al. [23], the free amino acids in the muscle were extracted with cold 80% (*v*/*v*) ethanol/water. Fresh tissue was homogenized with 20 mL of cold 80% ethanol using an Ultraturrax homogenizer (IKA, Staufen, Germany). The content of amino acids was analyzed with an automatic amino acid analyzer (HITACHI, L-8900, Tokyo, Japan).

### 2.8. Statistical Analysis

Data were presented as mean values ± standard error of mean (s.e.m). The statistical analyses were performed using SPSS 16.0 (SPSS Inc., 2005, Chicago, IL, USA). The normality and homogeneity of variances among groups were tested, and analyzed using the independent sample *t*-test (*t*-Student). Differences were considered significant when *p* < 0.05.

## 3. Results

### 3.1. Effect of the pEGFP-N1-GSK-3β Vector on the mRNA Expression of GSK-3β, β-Catenin, C/EBPα, and PPARγ in the Muscle of Zebrafish

**Figure 2:**
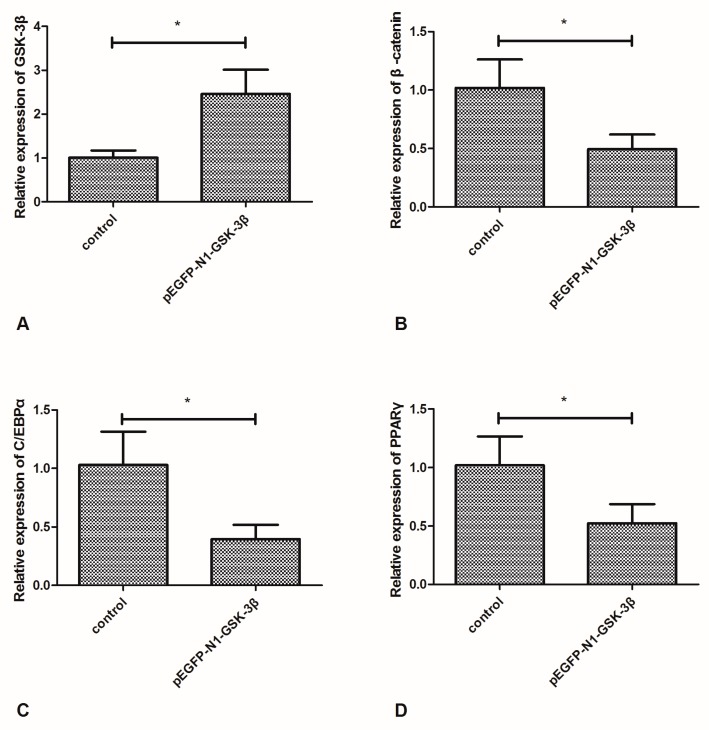
Effect of the pEGFP-N1-GSK-3β vector on the mRNA expression of GSK-3β, β-catenin, C/EBPα, and PPARγ in the muscle of zebrafish. (**A**) GSK-3β; (**B**) β-catenin; (**C**) C/EBPα; (**D**) PPARγ. Values are expressed as means ± s.e.m. (*n* = 6). Statistically significant differences are denoted by asterisk (*p* < 0.05).

Compared to the control group, the mRNA expression of GSK-3β was significantly increased by the pEGFP-N1-GSK-3β vector in the muscle of zebrafish (Figure 2A). However, the mRNA expression of β-catenin was significantly decreased by the pEGFP-N1-GSK-3β vector (Figure 2B). Moreover, the mRNA expression of C/EBPα and PPARγ was significantly decreased by the pEGFP-N1-GSK-3β vector (Figure 2C,D). 

### 3.2. Effect of the pEGFP-N1-GSK-3β Vector on the mRNA Expression of FAS, ACC, ACL, and HMGCR in the Muscle of Zebrafish

**Figure 3:**
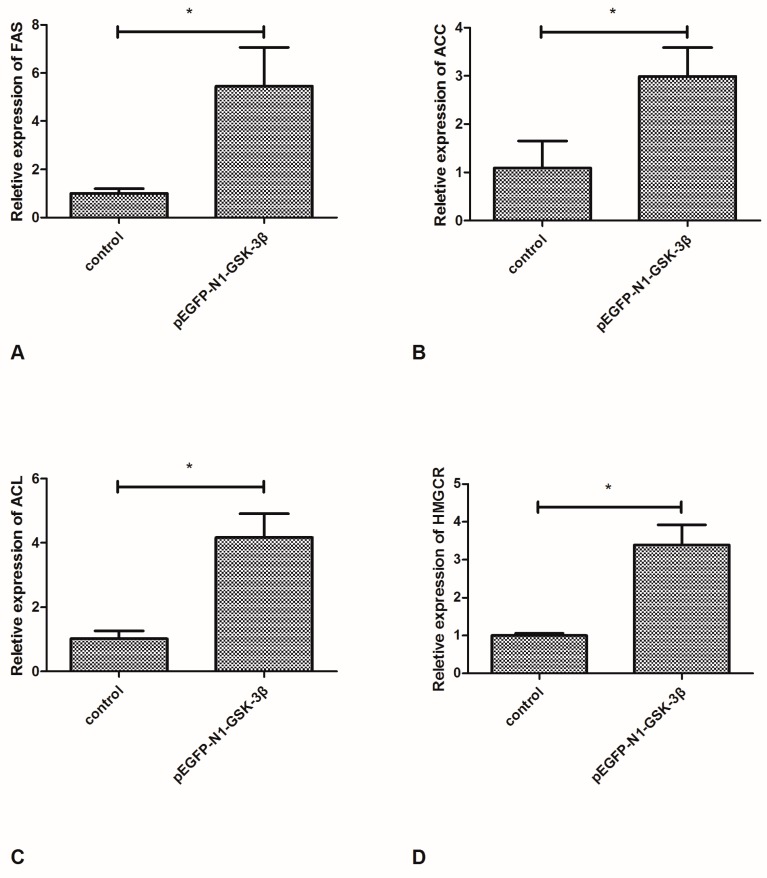
Effect of the pEGFP-N1-GSK-3β vector on the mRNA expression of FAS, ACC, ACL, and HMGCR in the muscle of zebrafish. (**A**) FAS; (**B**) ACC; (**C**) ACL; (**D**) HMGCR. Values are expressed as means ± s.e.m. (*n* = 6). Statistically significant differences are denoted by asterisk (*p* < 0.05).

Compared to the control group, the mRNA expression of FAS and ACC was significantly increased by the pEGFP-N1-GSK-3β vector in the muscle of zebrafish (Figure 3A,B). In addition, the mRNA expression of ACL and HMGCR was significantly increased by the pEGFP-N1-GSK-3β vector (Figure 3C,D).

### 3.3. Effect of the pEGFP-N1-GSK-3β Vector on the Activity of GSK-3β, FAS, ACC, ACL, and HMGCR in the Muscle of Zebrafish

**Figure 4:**
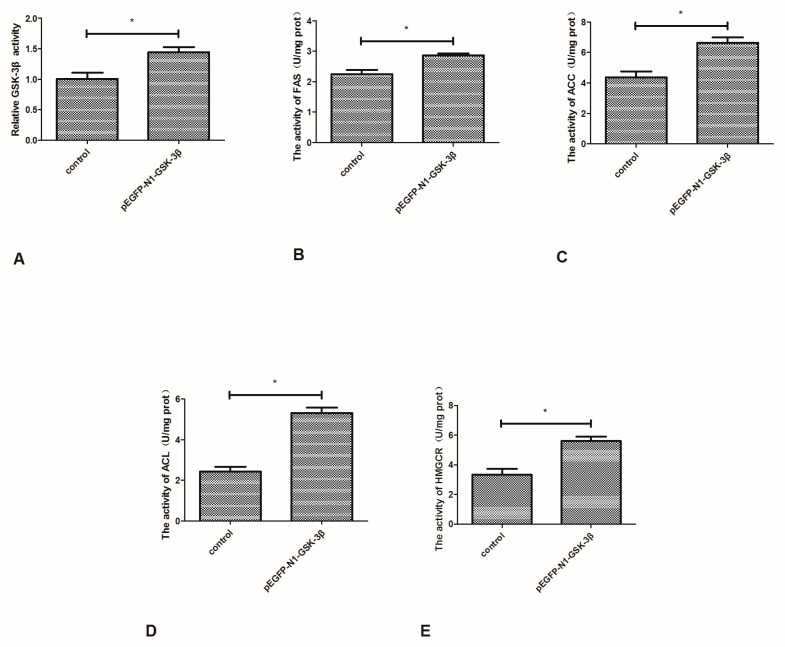
Effect of the pEGFP-N1-GSK-3β vector on the activity of GSK-3β, FAS, ACC, ACL, and HMGCR in the muscle of zebrafish. (**A**) GSK-3β; (**B**) FAS; (**C**) ACC; (**D**) ACL; (**E**) HMGCR. Values are expressed as means ± s.e.m. (*n* = 6). Statistically significant differences are denoted by asterisk (*p* < 0.05).

Compared to the control group, the activity of GSK-3β was significantly increased by the pEGFP-N1-GSK-3β vector in the muscle of zebrafish (Figure 4A). The activity of FAS and ACC was significantly increased by the pEGFP-N1-GSK-3β vector (Figure 4B,C). In addition, the activity of ACL and HMGCR was significantly increased by the pEGFP-N1-GSK-3β vector (Figure 4D,E). 

### 3.4. Effect of the pEGFP-N1-GSK-3β Vector on the Protein Expression of GSK-3β, β-Catenin, PPARγ, C/EBPα, and FAS in the Muscle of Zebrafish

**Figure 5:**
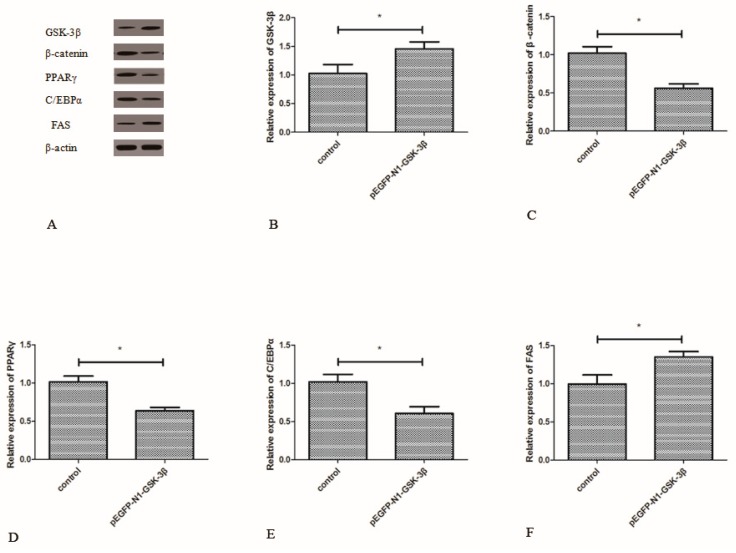
Effect of the pEGFP-N1-GSK-3β vector on the protein expression of GSK-3β, β-catenin, PPARγ, C/EBPα, and FAS in the muscle of zebrafish. (**A**) The protein bands; (**B**) GSK-3β; (**C**) β-catenin; (**D**) PPARγ; (**E**) C/EBPα; (**F**) FAS. Values are expressed as means ± s.e.m. (*n* = 6). Statistically significant differences are denoted by asterisk (*p* < 0.05).

Compared to the control group, the protein expression of GSK-3β was significantly increased by the pEGFP-N1-GSK-3β vector in the muscle of zebrafish (Figure 5B). The protein expression of β-catenin, PPARγ, and C/EBPα was significantly decreased by the pEGFP-N1-GSK-3β vector (Figure 5C–E). However, the protein expression of FAS was significantly increased by the pEGFP-N1-GSK-3β vector in the muscle of zebrafish (Figure 5F).

### 3.5. Effect of the pEGFP-N1-GSK-3β Vector on the mRNA Expression of TSC2, mTOR, S6K1 and 4EBP1 in the Muscle of Zebrafish

**Figure 6:**
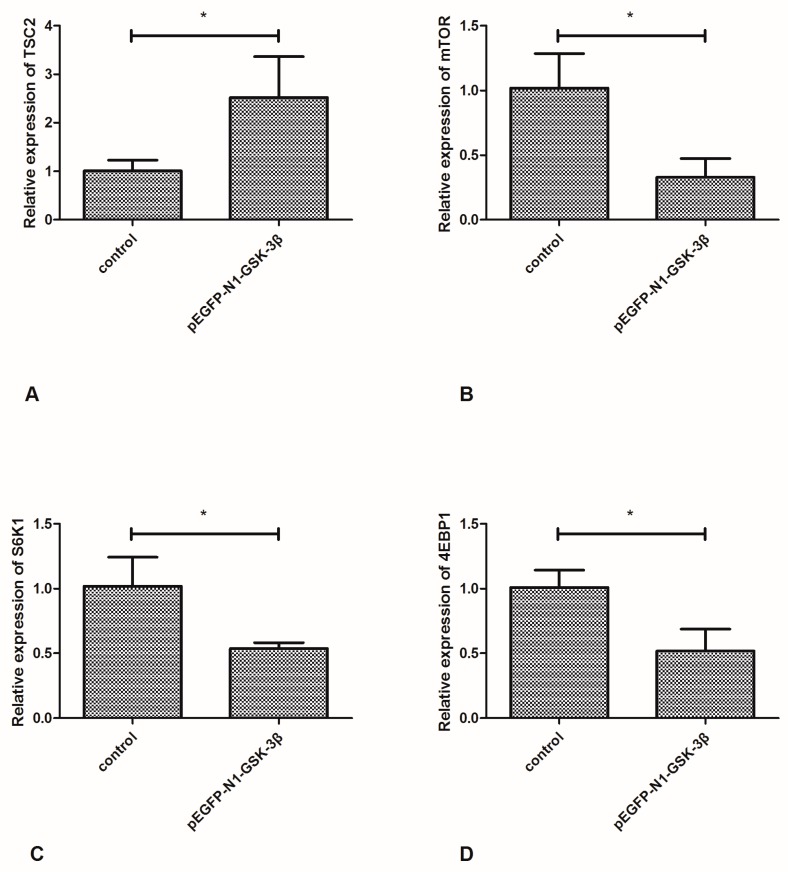
Effect of the pEGFP-N1-GSK-3β vector on the mRNA expression of TSC2, mTOR, S6K1, and 4EBP1 in the muscle of zebrafish. (**A**) TSC2; (**B**) mTOR; (**C**) S6K1; (**D**) 4EBP1. Values are expressed as means ± s.e.m. (*n* = 6). Statistically significant differences are denoted by asterisk (*p* < 0.05).

Compared to the control group, the mRNA expression of TSC2 was significantly increased by the pEGFP-N1-GSK-3β vector (Figure 6A). However, the mRNA expression of mTOR was significantly decreased by the pEGFP-N1-GSK-3β vector (Figure 6B). After GSK-3β RNA was overexpressed in the muscle, the mRNA expression of S6K1 and 4EBP1 was significantly decreased (Figure 6C,D).

### 3.6. Effect of the pEGFP-N1-GSK-3β Vector on the Protein Expression of TSC2, mTOR, S6K1, and 4EBP1 in the Muscle of Zebrafish

**Figure 7:**
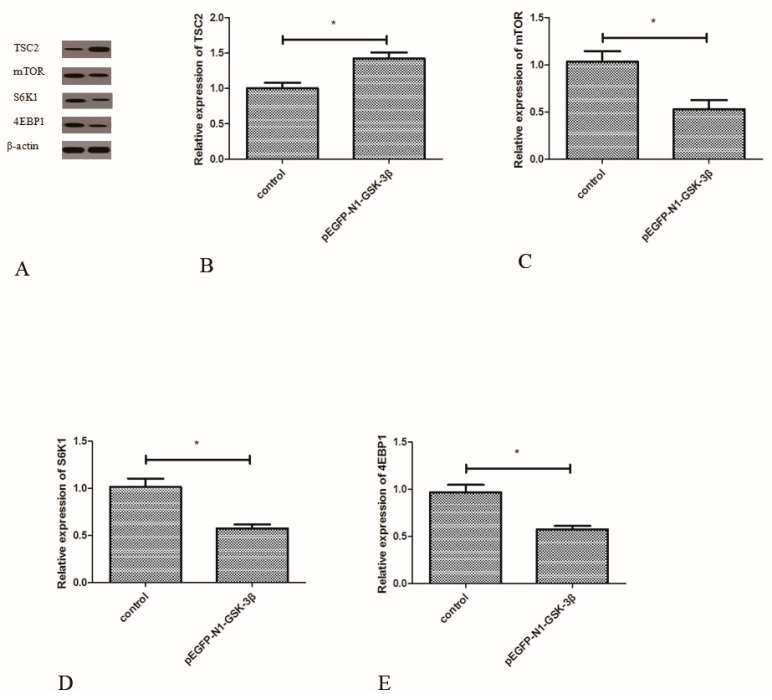
Effect of the pEGFP-N1-GSK-3β vector on the protein expression of TSC2, mTOR, S6K1, and 4EBP1 in the muscle of zebrafish. (**A**) The protein bands; (**B**) TSC2; (**C**) mTOR; (**D**) S6K1; (**E**) 4EBP1. Values are expressed as means ± s.e.m. (*n* = 6). Statistically significant differences are denoted by an asterisk (*p* < 0.05).

Compared to the control group, the protein expression of TSC2 was significantly increased by the pEGFP-N1-GSK-3β vector (Figure 7B). However, the protein expression of mTOR was significantly decreased by the pEGFP-N1-GSK-3β vector (Figure 7C). After GSK-3β RNA was over-expressed in the muscle, the protein expression of S6K1 and 4EBP1 was significantly decreased (Figure 7D,E).

### 3.7. Effect of the pEGFP-N1-GSK-3β Vector on the Levels of TG, TC, and NEFA in the Muscle of Zebrafish

**Figure 8:**
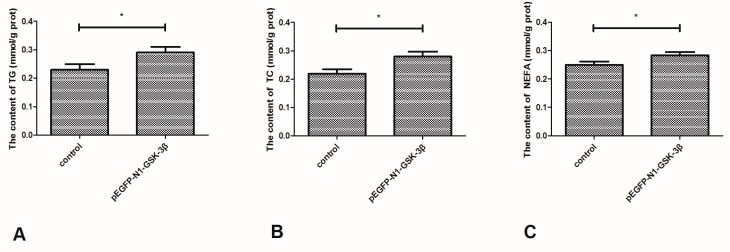
Effect of the pEGFP-N1-GSK-3β vector on the levels of TG, TC, and NEFA in the muscle of zebrafish. (**A**) TG; (**B**) TC; (**C**) NEFA. Values are expressed as means ± s.e.m. (*n* = 6). Statistically significant differences are denoted by an asterisk (*p* < 0.05).

As shown in Figure 8, the content of TG and TC was significantly increased by the pEGFP-N1-GSK-3β vector (Figure 8A,B). Furthermore, the content of NEFA was significantly increased by the pEGFP-N1-GSK-3β vector in the muscle of zebrafish (Figure 8C).

### 3.8. Effect of the pEGFP-N1-GSK-3β Vector on the Content of Free Amino Acids in the Muscle of Zebrafish

The content of free amino acids Arg, Lys, His, Phe, Tyr, Leu, Ile, Met, Val, and Thr in the muscle of zebrafish was significantly decreased by treatment with the pEGFP-N1-GSK-3β vector (Table 3).

## 4. Discussion

It is known that mTOR is a central controller of cell growth in response to cellular energy, nutrient levels, and growth factors [24]. In a previous study, GSK-3β was involved in regulating TSC2/mTOR signaling, which further controls glucose uptake and cell growth [18,19]. As an attractive target for mTOR signaling regulation, the activity of GSK-3β plays a negative role in controlling TSC2/mTOR signaling [25]. In addition, TSC2 antagonizes the mTOR signaling pathway via stimulation of GTP hydrolysis of Rheb and inhibits cell proliferation and cell growth [24]. In this study, both the expression and activity of GSK-3β were increased with injection of the pEGFP-N1-GSK-3β vector. The expression of TSC2 was significantly increased, but the expression of mTOR was significantly decreased by GSK-3β gene overexpression. The result of GSK-3β gene overexpression indicated that mTOR signaling could be inhibited by GSK-3β in the muscle of zebrafish.

In the present study, the expression of S6K1 and 4EBP1 was significantly decreased by the pEGFP-N1-GSK-3β vector in the muscle of zebrafish. It has been found that mammalian cell size was controlled by mTOR and its downstream targets S6K1, 4EBP1 and eIF4E [26]. S6K1, 4EBP1, and mTORC1-dependent pathways participated in regulating protein synthesis in skeletal and cardiac muscle and visceral tissues of neonatal pigs [27]. The expression of S6K1 and 4EBP1 was significantly decreased and mTOR inhibited by the pEGFP-N1-GSK-3β vector in the muscle of zebrafish, thus indicating that S6K1 and 4EBP1 were involved in regulating protein synthesis via mTOR signaling in the muscle of zebrafish.

Free amino acid content plays a significant role in the process of protein synthesis [28,29]. The size and composition of the free amino acid pool reflect the availability of circulating amino acids (Davis and Fiorotto, 2009) [30]. In the skeletal muscle, amino acids are the signaling regulators for protein deposition (Wu, 2013) [31]. Amino acids can stimulate protein synthesis, and the availability of amino acids affects the leucine stimulation of protein synthesis (Davis. 2002; Escobar, 2007) [32,33]. In this study, the content of free amino acids Arg, Lys, His, Phe, Tyr, Leu, Ile, Met, Val, and Thr in the muscle of zebrafish was significantly decreased by treatment with the pEGFP-N1-GSK-3β vector. Free amino acid content decreased due to GSK-3β overexpression and resulted in less free amino acids in the muscle, thus showing that GSK-3β could inhibit protein synthesis in the muscle of zebrafish. 

In Wnt signaling, the level of β-catenin is closely related to the activity of GSK-3β [34,35]. In this study, the expression and activity of GSK-3β was increased with injection of the pEGFP-N1-GSK-3β vector. The overexpression of the GSK-3β gene significantly decreased the mRNA and protein expression of β-catenin. These results indicated that GSK-3β inhibited β-catenin signaling by inhibiting the expression of β-catenin in the muscle of zebrafish. 

It is known that lipid is easily accumulated in the muscle and liver of fish [36,37]. FAS is one of the main lipogenic enzymes producing fatty acids, and the released fatty acids are accumulated in the lipid droplets of adipocytes [38]. In this study, the mRNA expression and activity of FAS, ACC, ACL, and HMGCR were increased by GSK-3β gene overexpression. This shows that GSK-3β may induce lipid deposition by increasing the expression and activity of FAS, ACC, ACL, and HMGCR. In addition, the content of TG, TC, and NEFA was increased by GSK-3β gene overexpression in the muscle of zebrafish. As HMGCR plays a key role in regulating TC generation, the increase of TC content may be related to the increase of HMGCR expression. 

Dietary and endogenous lipids are transported from the liver to the peripheral tissues for usage [39]. In a previous study, β-catenin was involved in regulating adipogenesis and lipid metabolism [40]. In diet-induced obesity, β-catenin regulates lipid metabolism and hepatic metabolic zonation [41]. To maintain lipid homeostasis, there is a balance between lipid deposition and transportation in the normal physiological status. As biochemical indexes of muscle were significantly affected by GSK-3β gene overexpression, GSK-3β may induce levels of TG, TC, and NEFA by regulating the activity of FAS, ACC, ACL, and HMGCR in the muscle of zebrafish.

A previous study found that transcription factors C/EBPα and PPARγ were involved in regulating the expression of genes relating to the proliferation of adipocytes [42]. In the adipocyte differentiation, C/EBPα and PPARγ induce the expression of various proteins and enzymes on lipid synthesis and deposition [43,44]. Furthermore, β-catenin signaling inhibits the expression of C/EBPα and PPARγ [45,46]. In this study, the expression of PPARγ and C/EBPα in muscle was inhibited by GSK-3β gene overexpression. The mechanism by which GSK-3β regulates lipid deposition in muscle may be different from that of adipocyte differentiation and proliferation. GSK-3β might regulate the expression of genes on lipid synthesis by decreasing PPARγ and C/EBPα or other transcription factors in the muscle of zebrafish, which needs to be further studied. 

## 5. Conclusions

**Figure 9:**
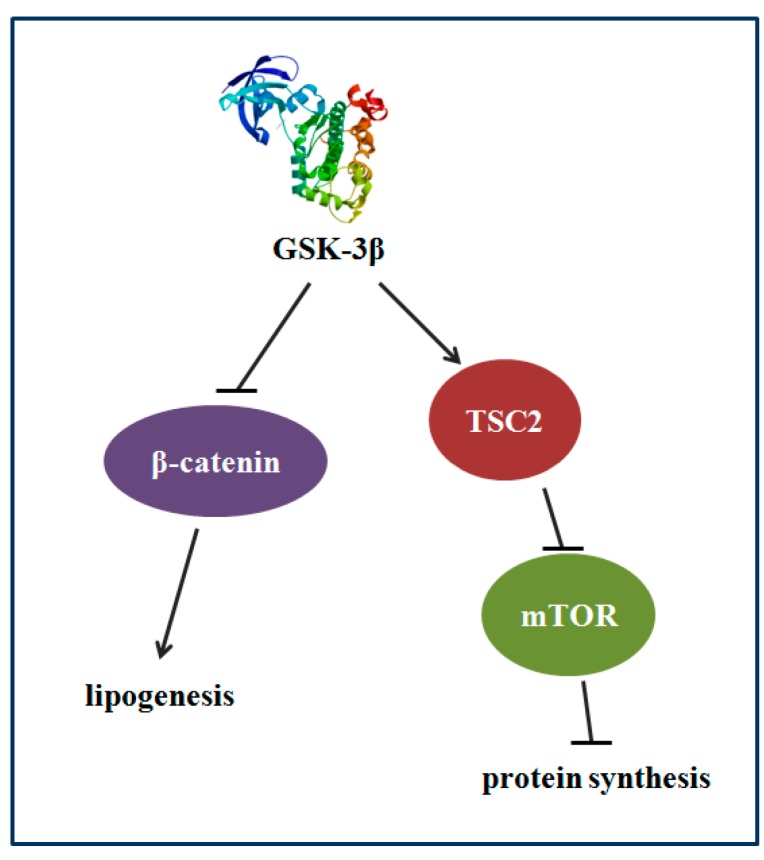
The proposed mechanism that GSK-3β participates in regulating protein synthesis and lipid deposition in zebrafish. GSK-3β may participate in regulating protein synthesis via TSC2/mTOR signaling and lipid deposition via β-catenin in the muscle of zebrafish.

The results of GSK-3β gene overexpression indicated that GSK-3β may participate in regulating protein synthesis via TSC2/mTOR signaling and lipid deposition via β-catenin signaling in the muscle of zebrafish (*Danio rerio*) (Figure 9).

## Figures and Tables

**Table 1:** Sequence of the primers used in this study.

Primer	Sequence (5′-3′)	Direction
GSK-3β-F1	CTGGTGAGCAGTAGGGTG	Forward
GSK-3β-R1	CGGATTCGTTCAAGACAA	Reverse
GSK-3β-F2	CCGGAATTCGCCACCATGTCCGGTCGGCCCAGAACC	Forward
GSK-3β-R2	CGGGGTACCCCGGTTGAGGTGTTAGCGGCGGAG	Reverse

*EcoR* I and *Kpn* I restriction enzyme sites are underlined.

**Table 2:** Real-time quantitative PCR primers for genes of zebrafish.

Target Gene	Forward (5′-3′)	Reverse (5′-3′)	GenBank
*GSK-3β*	TCTGCTCACCGTTTCCTTTC	CTCCGACCCACTTAACTCCA	NM_131381.1
*TSC2*	TGGCCTCTCTCAGCTCTCTC	CAGGTGAAACAGACGCCATA	NM_001328401.1
*mTOR*	TTGAATTTGAAGGCCACTCC	TAAGCAGACGTGAGCAGGTG	NM_001077211.2
*S6K1*	CATTTAGGTTTGCAGCACCA	AGTTGGCAGCTCTTCCAGTC	EF373681.1
*4EBP1*	AACGGACAAGGTGCAAAGAC	GTGGTTGGAATTGCCTGACT	NM_199645.1
*β-catenin*	GGAGCTCACCAGCTCTCTGT	TAGCTTGGGTCGTCCTGTCT	NM_001001889.1
*CEBP/α*	CACAACAGCTCCAAGCAAGA	AATCCATGTAGCCGTTCAGG	BC063934.1
*PPARγ*	CTGGACATCAAGCCCTTCTC	AGCTGTACATGTGCGTCAGG	NM_131467.1
*FAS*	ACAATGCTGGTGACAGTGGA	TACGTGTGGGCAGTCTCAAG	XM_009306806.2
*ACC*	AGGTGGTACGGATGGCTGCTC	GACGGTGCTGGACGCTGTTG	NM_001271308.1
*ACL*	AGACCTGATCTCCAGCCTCACATC	ATGCCACTGTCGAATGCCTTACTG	BC076484.1
*HMG* *CR*	ACGTCATCGGTTACATGCCAGTTC	GCCTTCAGTTGTCGCCATCGG	NM_001079977.2
*β-actin*	CCGTGACATCAAGGAGAAGC	TACCGCAAGATTCCATACCC	AF057040.1

**Table 3:** Effect of the pEGFP-N1-GSK-3β vector on the content of free amino acids in the muscle of zebrafish.

Essential Amino Acids	Control (μg/g Wet Weight)	pEGFP-N1-GSK-3β Vector (μg/g Wet Weight)
Arg	26.71 ± 0.46	24.22 ± 0.67 *
Lys	133.92 ± 1.38	130.43 ± 0.97 *
His	147.04 ± 2.36	139.47 ± 0.75 *
Phe	12.21 ± 0.57	10.77 ± 0.55 *
Tyr	38.77 ± 0.61	37.07 ± 0.81 *
Leu	27.57 ± 1.17	24.32 ± 0.37 *
Ile	12.70 ± 0.27	11.67 ± 0.57 *
Met	9.12 ± 0.43	8.02 ± 0.38 *
Val	38.38 ± 1.06	36.13 ± 0.31 *
Thr	146.92 ± 2.10	140.12 ± 3.62 *

Values are expressed as means ± s.e.m. (*n* = 6). Statistically significant differences are denoted by an asterisk (*p* < 0.05).
